# Software-Tool Support for Collaborative, Virtual, Multi-Site Molecular Tumor Boards

**DOI:** 10.1007/s42979-023-01771-8

**Published:** 2023-04-27

**Authors:** Matthieu-P. Schapranow, Florian Borchert, Nina Bougatf, Hauke Hund, Roland Eils

**Affiliations:** 1 Hasso Plattner Institute for Digital Engineering, University of Potsdam, Prof.-Dr.-Helmert-Str. 2-3, 14482 Potsdam, Germany; 2 Department of Radiation Oncology, Heidelberg Ion-Beam Therapy Center (HIT), Heidelberg University Hospital, Im Neuenheimer Feld 450, 69120 Heidelberg, Germany; 3 GECKO Institute, Heilbronn University of Applied Sciences, Max-Planck-Straße 39, 74081 Heilbronn, Germany; 4 Center for Digital Health, Berlin Institute of Health and Charité Universitätsmedizin Berlin, Kapelle-Ufer 2, 10117 Berlin, Germany

**Keywords:** Collaborative, Virtual, Multi-site, Molecular tumor board, Personalized medicine, Clinical process, Software tool

## Abstract

The availability of high-throughput molecular diagnostics builds the foundation for Molecular Tumor Boards (MTBs). Although more fine-grained data is expected to support decision making of oncologists, assessment of data is complex and time-consuming slowing down the implementation of MTBs, e.g., due to retrieval of the latest medical publications, assessment of clinical evidence, or linkage to the latest clinical guidelines. We share our findings from analysis of existing tumor board processes and defininion of clinical processes for the adoption of MTBs. Building on our findings, we have developed a real-world software prototype together with oncologists and medical professionals, which supports the preparation and conduct of MTBs and enables collaboration between medical experts by sharing medical knowledge even across the hospital locations. We worked in interdisciplinary teams of clinicians, oncologists, medical experts, medical informaticians, and software engineers using design thinking methodology. With their input, we identified challenges and limitations of the current MTB approaches, derived clinical process models using Business Process and Modeling Notation (BMPN), and defined personas, functional and non-functional requirements for software tool support. Based on it, we developed software prototypes and evaluated them with clinical experts from major university hospitals across Germany. We extended the Kanban methodology enabling holistic tracking of patient cases from “backlog” to “follow-up” in our app. The feedback from interviewed medical professionals showed that our clinical process models and software prototype provide suitable process support for the preparation and conduction of molecular tumor boards. The combination of oncology knowledge across hospitals and the documentation of treatment decision can be used to form a unique medical knowledge base by oncologists for oncologists. Due to the high heterogeneity of tumor diseases and the spread of the latest medical knowledge, a cooperative decision-making process including insights from similar patient cases was considered as a very valuable feature. The ability to transform prepared case data into a screen presentation was recognized as an essential feature speeding up the preparation process. Oncologists require special software tool support to incorporate and assess molecular data for the decision-making process. In particular, the need for linkage to the latest medical knowledge, clinical evidence, and collaborative tools to discuss individual cases were named to be of importance. With the experiences from the COVID-19 pandemic, the acceptance of online tools and collaborative working is expected to grow. Our virtual multi-site approach proved to allow a collaborative decision-making process for the first time, which we consider to have a positive impact on the overall treatment quality.

## Introduction

**Fig. 1:**
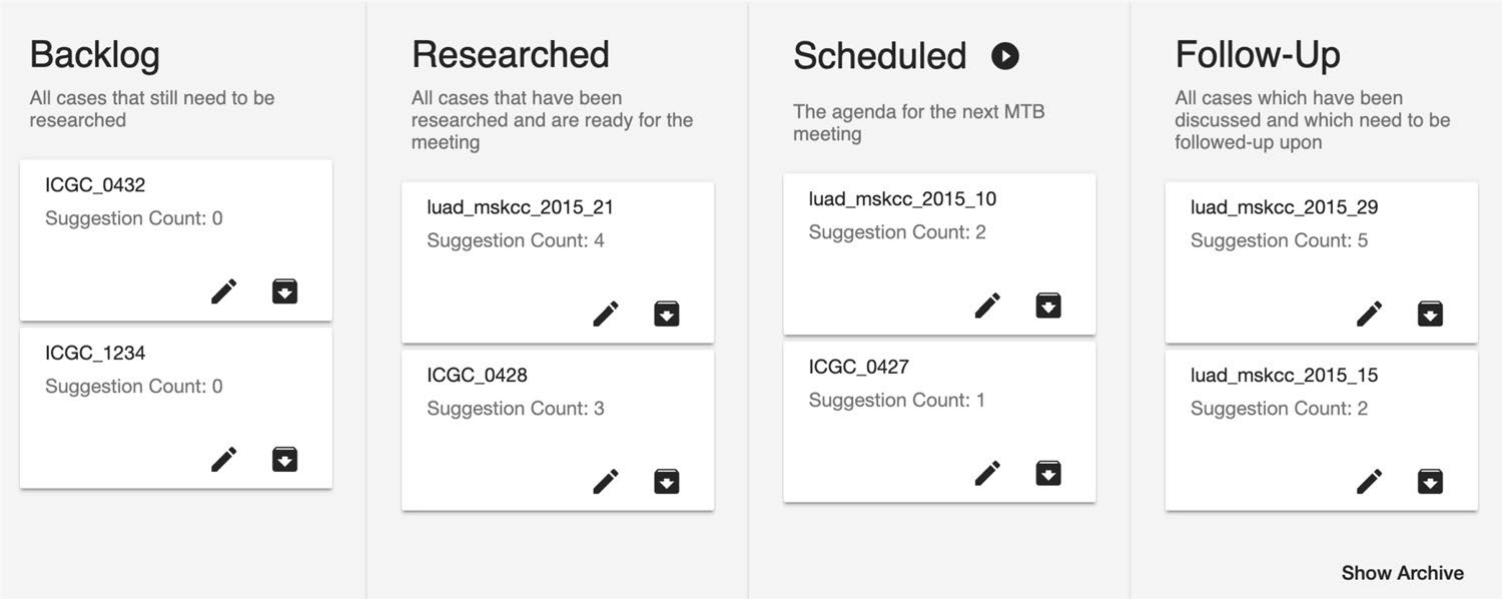
Several patient cases arranged in the MTB dashboard using Kanban methodology. From left to right: The backlog contains non-annotated cases, followed by already researched molecular profiles, cases scheduled for an upcoming MTB, and those that require a follow-up. Each card represents an individual patient case

The heterogeneity of cancer diseases demands collaboration between experts from multiple disciplines, e.g. medicine, biology, genetics, statistical data analysis, and bioinformatics [1, 2]. The wide adoption of Tumor Boards (TBs) as the de facto standard for oncology decision-making shows the importance of joint medical expertise. In TB sessions, oncologists come together on a regular basis to discuss every patient case individually per hospital. Jointly, available data are accessed and treatment recommendations proposed by the treating oncologists are discussed to derive an individual treatment plan per patient [3, 4]. In our work, we focus on Molecular Tumor Boards (MTBs), i.e. a specific form of TBs, which involve complex molecular data of the patient tumor as an additional basis for treatment recommendations [4].

Thanks to the latest advances in medical devices and hardware, more and more fine-grained data can be acquired in a shorter time. For example, the adoption of Next-Generation Sequencing (NGS) for molecular screening of tumors becomes increasingly affordable whilst making fine-grained molecular data available [5]. At the same time, it is getting more and more complex for oncologists to gain insights from this sheer amount of data due to the lack of adequate tool support and access to the latest molecular findings. As a result, the acquisition of diagnostic data is no longer a bottleneck, but rather its analysis and interpretation.

Therefore, we follow the hypothesis that the use of adequate software tools can help to reduce time required to prepare and perform TB sessions whilst enabling the use of more and more individual molecular markers for medical decision-making. Experiences from working with online and video conferencing tools during the COVID-19 pandemics showed that cooperation between experts from different hospitals and disciplines is feasible, and the actual physical location of a person is no longer a limiting factor. Therefore, we believe that the structured acquisition and management of oncology data is a key-enabler for evidence-based medicine as it builds the foundation for cooperative sharing of medical knowledge between oncologists even beyond hospital and country borders.

In an interdisciplinary approach, we brought together subject-matter experts with oncology, medical, and software engineering expertise. We followed an engineering approach to observe the current state-of-the-art implementation of TBs in German hospitals. We share reported challenges and limitations of current TB implementations and derived specific requirements for software tool support by performing software requirements engineering [6].

We observed that TBs and MTBs among hospitals vary due to missing standardization, and, to the best of our knowledge, no standardized process models exist in the literature. Today, tools for MTB support can be divided into two categories: a) Clinical Decision Support Systems (CDSS) and b) management support systems. CDSS focus on the preparation process and either leverage Artificial Intelligence (AI) to extract relevant information or store expert experiences such as variant-treatment combinations [7]. Annotation and publication databases play a crucial role in the preparation phase, but needs to be queried individually. Online services and cancer variant interpretation tools aim to link medical publications and data from disease-specific databases to support the information-seeking process for researchers and clinicians [8, 9]. In contrast, the literature rarely reports management support systems, i.e., tools supporting look-up of patient data, tracking of case progress along the tumor board process, creating presentations, documentation, and follow-up of patients. In the context of our work, we developed a software prototype MTB Assist for collaborative, virtual, multi-site MTBs as depicted in Fig. 1. It builds the basis for the integration of our proposed clinical MTB process and its evaluation together with medical experts [10, 11].

The given results were created in the context of the HiGHmed consortium, which is one out of four nationally funded consortia of the German Medical Informatics Initiative (MII) [12]. The MII is a strategic research program funded by the German Federal Ministry of Education and Research, which aims to improve the use and exchange of data between hospitals, medical doctors and researchers to improve existing and create new innovative clinical processes [13]. Amongst others, the HiGHmed consortium consists of the medical sites: Heidelberg University Hospital, University Medical Center Göttingen, Hannover Medical School, University Hospital Schleswig-Holstein, University Hospital Cologne, University Hospital Würzburg, Charité Universitätsmedizin Berlin, University of Münster, and the German Cancer Research Center in Heidelberg. Our contribution builds on the expertise of medical experts from all HiGHmed partners [14]. As a result, we believe that our findings can serve as a basis for a national MTB strategy.

The remainder of the work is structured as follows: After setting our work in the context of related work in Sect. “Related Work”, methods of our engineering approach are described in Sect. “Methods”. In Sect. “Requirements Engineering”, we share the results of the conducted requirements engineering process followed by our contributions in Sect. “Contributions”. We evaluate our findings in Sect. “Evaluation” and discuss them in Sect. “Discussion and Limitations”. Our work concludes with an outlook in Sect. “Conclusion and Outlook”.

## Related Work

Nowadays, MTBs are being established increasingly in hospitals and cancer care centers [3, 4, 15–17]. They are often part of clinical studies, e.g. the MASTER program in Heidelberg [18]. Although MTBs are of growing importance for the improved care of cancer patients in clinical practice, there is currently no common definition of and standard for implementing them available [5, 19, 20]. As a result, the implemented clinical processes for preparation and conducting of MTBs vary across hospitals, which impacts the reproducibility and quality of MTB findings [21]. To the best of the authors’ knowledge, only informal descriptions of MTB implementations were published at the time of writing, e.g. describing individual experiences of MTB implementation per hospital, which are neither targeted to be standardized for multi-site use nor incorporate dedicated software tool support [4, 22, 23]. Even though more comprehensive descriptions of software systems for supporting MTBs have been published, there has been given little emphasis on the modeling and support of underlying clinical processes [11, 24].

For the first time, we define in Sect. “Clinical Process Model” a formal description of the clinical process for implementation of virtual, multi-site MTBs building upon the standardized Business Process and Modeling Notation (BPMN). Our findings summarize our conducted multi-disciplinary work together with subject-matter experts from leading oncology centers and hospitals across Germany. We believe that our results can serve as a blueprint for standardized implementation in hospitals worldwide. Furthermore, we share details about our software tool in Sect. “Software Prototype”. It is designed for oncologists assisting them during the implementation of the individual clinical process steps: preparation, conducting, and follow-up of MTBs. It makes use of data analysis methods combining case-specific data, annotation data from the latest international publications, and specific annotation by oncology experts. As a result, our software tool provides a technical proof-of-concept for sharing oncology knowledge across multiple centers comparable to exiting tools already established in other disciplines, e.g. StackOverflow [25]. Apart from common meeting tools such as projectors, video conferencing software for collaborations or MS Office software for documenting and presenting cases, dedicated software tools for preparation, conducting meetings, documentation, and follow-up are not reported in the literature [26–28].

Nowadays, case data used for MTBs in clinical settings is often stored across distributed data silos, e.g., Electronic Health Records (EHRs), Hospital Information Systems (HISs), Laboratory Information Management Systems (LIMSs), or local workstations. However, such distributed data acquisition and storage are a blocker for collaborative real-time data access. It makes manual data aggregation and analysis necessary, which binds clinical staff and is considered to be time-consuming [29, 30]. Our software tool combines relevant clinical data provided by the Medical Data Integration Centers (MeDICs) within HiGHmed [14]. Especially, the ongoing COVID-19 pandemics had a huge impact on the clinical routine. One positive effect was the early adoption of video and telephone conference tools to enable virtual collaboration independently from the physical location whilst protecting personnel from potential infection hotspots [31, 32].

From our observations, MTB software tools can categorized as (a) Clinical Decision Support Systems (CDSSs) and (b) management support systems.

First, CDSSs aim to support mainly the preparation process, e.g. through the extraction of relevant medical information from international knowledge bases such as CIViC, OncoKB and cBioPortal [7]. Although annotation databases and publication search engines, such as PubMed, EMBASE and ASCO, play a key role in the preparation phase, they need to be queried manually, one after another, by the clinical expert. So far, selected online services aim to link medical publications and data from disease-specific databases to support the information retrieval process for researchers and clinicians [33]. Other services provide curated cancer-specific knowledge to aid oncologists during the preparation of MTBs or to support therapies selection [34, 35]. However, we consider our software not as an example for a CDSS, but as an application that makes use of existing CDSSs to improve the manual data annotation process.

Examples of management support systems are barely reported in the literature. Typical management tasks include case creation, look-up of case data, tracking of case progress along the tumor board process, creation of presentations, documentation, and follow-up of cases. One example is OncoLens used for managing TBs at the DeKalb Medical Cancer Center [36]. Its main focus lies on optimizing TB preparation time and to allow care providers to share and discuss treatment plans for cancer patients in real time. Another example is NAVIFY, which enables the management of TBs from data ingestion to documentation, notably reviewing radiology and pathology data [37, 38]. Therefore, we assign our software tool to the category management support systems for oncology. In addition to the aforementioned capabilities, our software tool also integrates external data sources in a modular way focusing on the specific requirements of MTBs. Our software tool builds the technical foundation of a multi-site database of participating hospitals, which contains—amongst others—point-of-care knowledge, case-specific annotations of individual molecular findings, treatment recommendations, and clinical studies. The data is enriched with relevant non-molecular data enabling the search for similar patient cases using numerous criteria, including clinical data, lab data, medication, or treatment recommendations. For further details, please refer to Sect. "Software Prototype".

## Methods

In the following, we share details about the incorporated methods and engineering approaches.

**Fig. 2:**
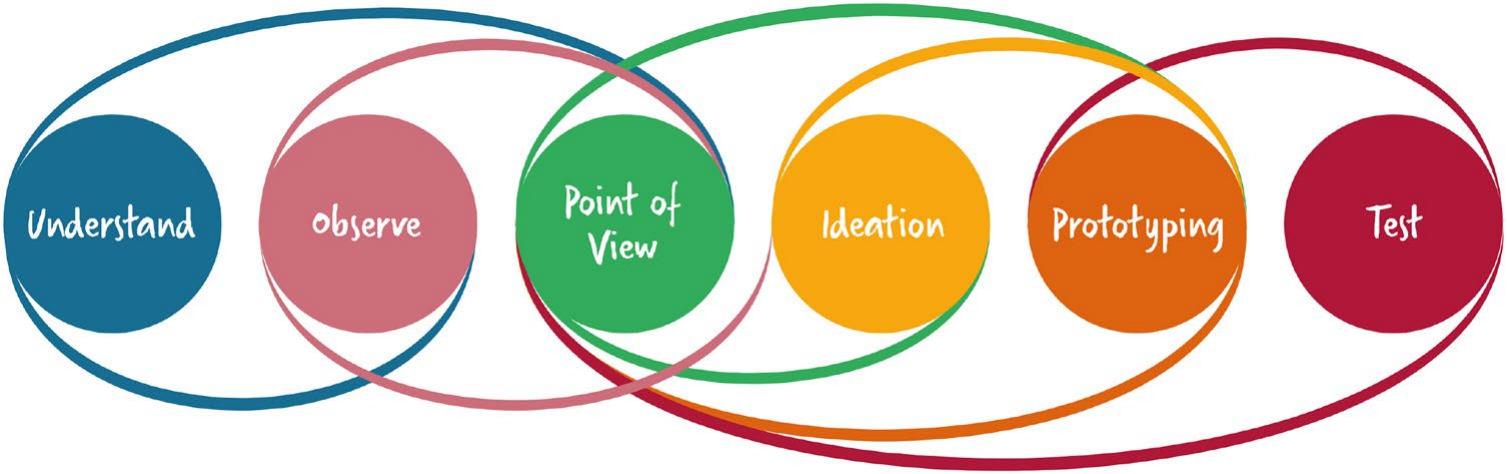
Design thinking process adapted from [39]. (1) We *understood* the problem space through semi-structured interviews with subject-matter experts (*n* = 8). (2) We *observed* the preparation and conduct of MTB sessions. (3) The *point of view* is defined through functional and non-functional requirements and clinical process models. (4) In the *ideation* phase, we drafted low-fidelity sketches and evaluated them with oncologists. (5) Based on the feedback, we implemented *prototypes* of our web application integrating regular user feedback. (6) User *tests* were performed with annotators (*n* = 2), adopting the think-aloud method

For our work, we followed an engineering approach that incorporated the Design Thinking (DT) methodology, outlined in Fig. 2. DT is a method to identify and solve problems incorporating multiple tools and steps, e.g. ideation, prototyping, and iterative testing phases [39]. In particular, we conducted interviews with subject-matter experts from university hospitals across Germany to identify current limitations and define a clinical process model for adopting MTBs in the clinical setting. Furthermore, we derived specific requirements for software tool support to facilitate virtual collaborations between oncologists through a shared knowledge base including also molecular-genetic data even across the borders of individual hospitals.

*Observations and Personas: * We visited MTBs of two major German university hospitals and interviewed subject-matter experts. The interviewees (*n* = 8) were identified through purposive sampling: they included translational oncologists, who routinely prepare MTB cases (*n* = 4), biomedical informatics experts from the German Cancer Research Center (*n* = 2), a molecular biologist (*n* = 1), and a project manager (*n* = 1).

After analysis of the interviews, we were able to identify the following three personas involved in the observed MTB processes: (a) the treating physician, (b) the MTB research expert, and (c) the moderator. Personas are an instrument to help identify specific user needs and build the foundation for requirements engineering [39, 40].

*Clinical Process Modeling: * Based on the identified needs per persona, we defined specific clinical process models for: (a) preparing, (b) conducting, and (c) follow-up on MTB sessions. We used the graphical Signavio Online Process Modeler to create BPMN version 2.0 process models as outlined in Sect. “Clinical Process Model” [41, 42]. Our process models were evaluated together with subject-matter experts during oncology meetings within the HiGHmed consortium.

*Rapid Prototyping: * Prototyping is a powerful way to evaluate ideas at an early stage, which aids in proving underlying assumptions [43]. For example, we drafted whiteboard sketches to align ideas and present iterative software prototypes to selected medical experts. Based on their feedback, we designed our software tool MTB Assist supporting oncologists in preparing, conducting and follow up of MTB sessions. MTB Assist is a Web application that allows easy access via Web browsers without local installations and configuration as part of the AnalyzeGenomes.com platform [8, 44].

The software consists of a Graphical User Interface (GUI) implemented in Angular 6, a backend server providing a RESTful API, and an In-Memory Database System (IMDB) enabling real-time data analysis and linkage, e.g. for population research and to support the follow-up process [45].

The GUI is designed to provide interactive system response time, which is a crucial property given the limited time for preparing and conducting TBs and the extensive interaction with the tool during these processes. Furthermore, the use of Angular for the GUI supports the design language Material Design, which ensures high-quality design and usability. The backend server is implemented in Python and incorporates the Python Flask framework [46]. It was selected due to its ability to build upon existing tools and maintain software features. The backend server handles requests from Angular’s data stores, assembles appropriate database queries, sends them to the IMDB, processes database results, and assembles information from the connected data stores.

*Evaluation: * MTB Assist was evaluated based on user tests with annotators (*n* = 2) from a major German cancer center with regular MTBs. The evaluation incorporated two iterations. Each test participant was first introduced to the purpose of the MTB support tool, then asked to simulate the whole MTB process. Along with the test, we adopted the Think-Aloud method to encourage the interviewees to talk about their impressions and actions [47].

## Requirements Engineering

In the following, we share our observations from our conducted DT understanding and observation phases and the derived requirements for adequate software tool support.

### Observations and Current Limitations

Today, the implementation of TBs and MTBs resp. varies from country to country and hospital to hospital due to the absence of a process standardization. In the following, we introduce our observed challenges denoted as C, which we acquired during our DT sessions conducted with subject-matter experts in a real-world clinical setting as outlined in Sect. “Methods”. Despite informal process descriptions from individual hospitals, detailed graphical process models do not exist in the literature to the best of our knowledge.

Nowadays, only selected oncology patients are discussed in an MTB session [48, 49]. Admitted patients have failed the standard-of-care cancer treatment regimen and fulfill other study-dependent eligibility criteria that are reviewed by a dedicated committee. Exemplary reasons for this selective procedure are sequencing costs, time constraints, and comparable high efforts for data analysis.

As soon as a patient has been admitted, a specific molecular analysis of the tumor is ordered by the treating physician, e.g. WES, WGS, tNGS or RNA-seq [4, 22, 27]. As a result, the MTB research expert receives a list of genetic variants, e.g. in the form of an MS Excel spreadsheet, to prepare the case discussion prior to the actual MTB session. Based on our observations, the number of MTB research experts ranges from one to three and also the qualifications range from part-time working assistant doctors to oncologists that are dedicated to this role. For each variant in the file that imposes a functional genetic change, potential medication options are researched by retrieving results denoted as *entries* from medical knowledge bases also known as *sources*, e.g. CIViC, PubMed, ASCOPubs, COSMIC, cBioPortal, VarSome and Mastermind [50–56].

For each case presented in an MTB session, individual treatment recommendations are researched from different medical knowledge databases based on a list of genetic variants [9]. The annotation includes druggability options, drug evidence levels, and ongoing clinical studies [18, 57]. This annotation process typically includes the variant, drug, the drug’s evidence level, and a reference to the source [18]. In addition, ongoing clinical studies are identified from sources such as ClinicalTrials.gov according to one interviewee [57].

#### Preparation of a Case for Discussion in an MTB Session

In the following, we assembled identified challenges that were observed during the preparation phase.

1. **Time-consuming, manual information retrieval:** Despite the increasing relevance of knowledge databases, the primary literature is always retrieved. The manual review of research articles from sources, such as PubMed, typically follows consecutive steps reported by one expert. First, different search term combinations are tested from general, e.g. "gene name", to more specific search terms, e.g. "gene name + affected protein". Titles of retrieved results are subsequently read and, if deemed relevant, the article abstracts are reviewed. If considered as relevant, specific sections of the article are read to decide whether it matches the genetic variant. The breadth of available sources results in the need to open a multitude of browser tabs to check the availability of entries. This way of information retrieval is tedious and may result in missed treatment recommendations.
2. **Identification of similar historic cases: **Annotations already evaluated in historic cases may be of help for a current case. Medical and treatment data from historic cases is considered as a valuable source for medical knowledge. However, we observed that annotations are stored in local, non-indexed files, which makes them difficult to search and retrieve.
3. **Limited access to local case data:** Intra- and inter-centric information exchange is restricted, an exchange of de-identified data is not possible, yet. Thus, available medical expertise is also limited.
4. **Limited resources for full analysis of molecular data:** Depending on the number of found genetic variants, the research work ranges from a few minutes to several work days, but this has to be restricted to a maximum of four hours to keep up with the number of cases that require analysis, according to one interviewee. Due to the time-intensive nature of research, the distribution of sources, the given time limitation per case and the current way of storing annotations, incorporating information from all available sources including historic cases during the annotation of a given variant is challenging.
5. **Relevance of molecular findings for treatment:** Not only data sources, also searching for each identified genetic variant is infeasible due to the possibly large number of variants and unspecified relevance regarding their pathogenicity. Due to the breadth of existing and non-harmonized knowledge bases and primary literature, information seeking is tedious and may result in missed treatment recommendations. Historical cases are a valuable source; however, those cases often are not indexed and are difficult to retrieve.
6. **Keeping track of status of individual patient cases:** In each MTB session, several patient cases are discussed, demanding to follow the previously outlined analysis process. Keeping track of the current preparation state, i.e. "needs to be researched", "is researched or annotated", "will be discussed" and "needs a follow-up" of each case is challenging. We found out that it is hard to keep track of the current preparation state because multiple cases are prepared by a single person in parallel.
7. **Presentation of treatment recommendations:** Once treatment recommendations are researched, presentation slides are assembled by copying results into a Microsoft PowerPoint slide deck. This process needs to be repeated for each patient and tumor board, which requires time that could be utilized to conduct further research.

Once the presentation is available, the preparation has ended and the case is prepared for discussion in an MTB session.

#### Conduct an MTB Session

In the following, we assembled identified challenges that were observed during the conduct of an MTB session.

MTBs are currently held on a regular basis, e.g., weekly or biweekly, along with a much larger number of indication-specific tumor boards, each conducted once or twice a week, depending on the number of cases per indication [16, 49]. Each meeting lasts 60–90 min, discussing approx. ten cases.

MTBs are conducted in a conference room with one or two large presentation canvases/screens and include external practitioners, who receive feedback on their cases. Each case is introduced by summarizing the patient’s medical history, then discussed. Finally, treatment suggestions are documented. A video conferencing system on the same or a separate screen may be used to connect to external physicians, who want to receive feedback on their cases. The meeting is moderated by the MTB research expert.

Each case is introduced summarizing the clinical anamnesis of the current case typically by the moderator. In a minority of the cases, radiological and pathological images and/or reports are retrieved and discussed.

All MTBs attendees must be given the possibility to prepare for each case to have rich discussions and fewer postponed meetings. Strong digital collaborations between the participants require differing permissions to a patient’s case sub-resources.

C8 **Case data stored across multiple data silos:** Often, multiple information systems are used to access data, highlighting the need for a unified interface. We observed in one tumor board that three different systems were used to access different types of data, which shows how clinical data is spread. The search for relevant data interrupts the meeting flow, contradicting that the focus of all participants shall be directed solely towards the discussion of the case. Whenever a question cannot be answered due to missing details, the case discussion is postponed to the next meeting adding a delay to treatment finding.
C9 **In-advance preparation for subject-matter experts:** Ultimately, the treatment recommendations are presented and briefly discussed by all attendees. Exemplarily, we measured the time per case in a selected MTB session, resulting in an average of 4:04 min with a minimum of 54 s and a maximum of 8:25 min. Brief meeting times of approx. one to two minutes typically indicate that there was an open question, which could not be answered directly due to missing information or data. In such cases, the discussion of the case is postponed to one of the upcoming sessions once the question documented by the moderator can be answered. We also observed that the discussion is mostly happening between the moderator and a single person, while most attendees listen only. Even though the knowledge is highly specialized and given a short time available to present each case, we assume that a better preparation of all participants before the meeting would create an enriched discussion environment and less postponed discussions.
C10 **Access rights and data privacy:** Stronger digital collaborations between the participants before or during the meeting entail that different access rights to parts of the case data must be provided.
C11 **Documentation of treatment recommendations:** After proposal of a treatment recommendation, results are documented. We observed that documentation is either directly written into free-text fields of a documentation system, e.g. the Gießener Tumordokumentationssystem (GTDS) or OnkoStar, or paper notes are digitized after the meeting [58, 59].

After the completed discussion of a patient case in the MTB session, treatment recommendations including suitable clinical studies are forwarded to the treating physician. Research experts are responsible for a periodic follow-up to find out whether treatment suggestions were applied and to generate statistics about how they affected patient outcomes. There is no established standard for follow-ups, which often involves multiple phone calls with cancer registries and treating oncologists.

### Software Requirements

Based on the analysis of our observations shared in Sect. “Observations and Current Limitations”, we derived the following list of functional software requirements (**F**) for a Web-based software support tool [60].

1. **Data privacy:** Only permitted users shall have access to case data and all patient-identifiable data shall be pseudonymized so that only treating physicians can establish the link to their patients (C10).
2. **Automatic import of case data:** Relevant case data, e.g., the clinical history, radiology, pathology reports as well as molecular data, shall be imported automatically from the respective HIS and displayed in a meaningful manner (C8).
3. **Visual clinical process overview:** Provide a graphical overview of the preparation progress, display a list of cases that will be discussed in the upcoming meeting, and cases that require a follow-up (C6).
4. **Variant annotation:** Enable users to create and save annotations of genetic variants into a database with a unified structure including entries, their sources, medication, evidence level, and an appropriate reasoning (C2, C3).
5. **Ranking of genetic variants:** Calculate a relevance score for individual genetic variants based on their pathogenicity (C4, C5).
6. **Parallel data extraction:** Inform about the availability of entries in annotation sources w.r.t. a genetic variant (C1, C4).
7. **Similar cases:** Identify similar cases based on the molecular genetic profile of the tumor (C2, C3).
8. **Collaboration:** Case information such as annotations shall be shareable to obtain feedback from peer researchers and clinicians (9).
9. **Case presentation:** Automate the process of meeting slide creation with a one-click solution containing relevant information per case to facilitate discussion during the MTB meeting (C7).
10. **Documentation:** Provide a documentation functionality for treatment recommendations linked to the case (C11).

In addition to these functional software requirements, we also identified the following non-functional requirements (**N**).

1. **Usability:** The software shall have a plain, non-overloaded design, being easy and intuitive to use by trained and non-trained users.
2. **Maintainability:** For the IT staff, it shall be easy to add new components, like data sources, and enhance existing functions, e.g. calculation of relevance scores, and roll-out updates or bug-fixes to all users in a timely manner.
3. **Minimal setup effort:** Medical professionals as users of the software should have minimum efforts to install, setup, and configure the software prior to use it.

## Contributions

In the following, we share our contributions, i.e., the clinical process models for conducting MTBs and the proposed software support prototype.

### Clinical Process Model

Together with subject-matter experts from oncology, we defined clinical process models for MTBs to support their adaption in clinical settings. The following process models aim to support the standardized implementation of MTBs in clinical settings.

#### MTB Preparation

**Fig. 3:**
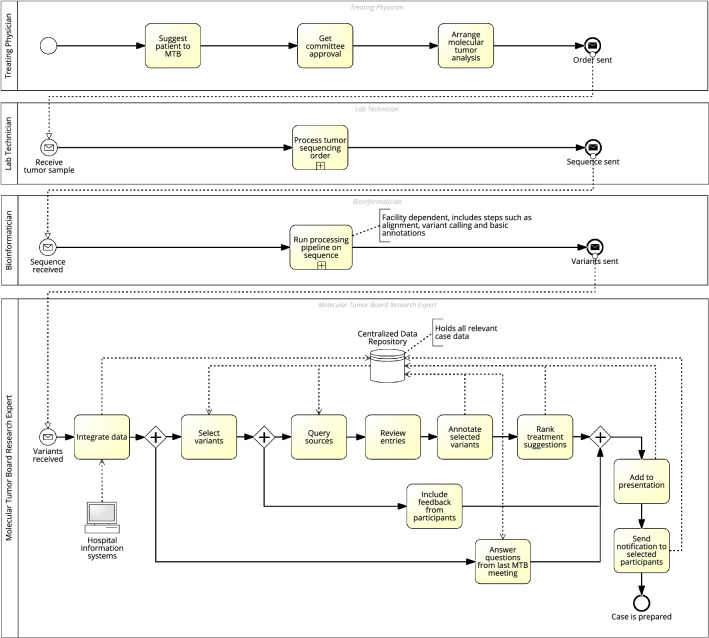
Process model for MTB preparation. For inclusion to an MTB, a patient must be approved. Approval may be given when state-of-the-art therapies have failed. Next, molecular analysis is ordered, and lab technicians sequence the tumor to retrieve the list of genetic variants. The research expert then creates a case in the MTB tool by merging all relevant case data from different sources. The identified genetic variants can be used to query similar cases. Variants are ranked based on their therapeutic relevance according to external sources like Var-Some. The research expert selects the most relevant variants and annotates them with identified therapies by literature review. Tool support is given by generating search queries automatically, leveraging named-entity recognition to highlight genes/drugs, and generating text summaries. The variant, drug, evidence level, source, and explanation are annotated to the case. Suitable therapy suggestions are ranked considering the medical history of the patient, the evidence level, and the feasibility of therapy. Additional information is drawn from historical cases to include existing clinical knowledge and improve preparation efficiency. The patient’s de-identified case data can be made available to other researchers. The patient is scheduled for one of the upcoming MTB meetings, and the presentation view including all necessary information can be started right away

The MTB preparation process per case is depicted in Fig. 3 and involves the following user roles: treating physician, lab technician, bioinformatician, and the MTB research expert. The latter role should consist of members from a scalable team of oncologists with a specialization in molecular biology, who are able to take both a clinical and a personalized medicine perspective.

The process is initiated by the treating physician or a study nurse, who suggests a patient for discussion in an MTB. Each registration needs to be approved by a committee that reviews whether state-of-the-art therapies have failed so far. With decreasing sequencing costs, growing knowledge about variant-disease-drug associations, quicker annotations and evolving guidelines, more and more patients will receive the opportunity to be presented in an MTB in the near future. We expect that molecular data will be part of all tumor boards soon. Upon approval, a molecular analysis targeted to the patient is ordered by the treating physician. The tumor is sequenced by lab technicians and a data processing pipeline is run by bioinformaticians, resulting in a list of genetic variants.

We assume the final output containing the genetic variants has a defined structure with a minimum set of required attributes as outlined in Sect. “Software Prototype”. This enables an interoperable integration into the centralized data repository. Further patient data such as radiology/pathology reports and clinical data shall be made accessible as well from the corresponding HIS. By the time the sequencing is done, the MTB research expert creates a case in our MTB tool by integrating all relevant patient data, i.e., ideally without the need to log in, search and retrieve data manually from the corresponding HIS or by opening files from a local hard drive.

Relevant clinical data sources including similar cases are queried w.r.t. identified variants. Furthermore, variants are ranked based on their therapeutic relevance according to scores obtained from data sources such as VarSome and functional effect prediction algorithms. Both steps may happen automatically through an assisting software. The research expert selects the most relevant variants, reviews the retrieved entries, and annotates them with identified therapies. Besides the variant, drug, evidence level, and source, an explanation is added to the annotation that may help other meeting participants and upcoming researchers to understand the reasoning of the research expert in preparation for the meeting, during the meeting or in retrospect. Among the annotations, suitable therapy suggestions are ranked considering the medical history of the patient, the evidence level, and the feasibility of therapy, e.g., by taking the location of clinical trial study centers into account.

Information is additionally drawn from historic cases gathered over time, optimally from multiple cancer centers. This allows to include existing clinical knowledge and improve preparation efficiency. During the preparation process, which includes annotation and treatment suggestion ranking, the research expert may include feedback from other attendees and researchers, by granting access to the patient’s de-identified case data. The research expert may also consult the expertise from other external research experts even if annotation databases remain local, i.e., are not interoperable and shared.

Compared to our observations outlined in Sect. “Observations and Current Limitations”, each patient case should be discussed a single time given the complete set of relevant data. We leave the option to postpone cases whenever new ideas were brought up by the participants that require further investigation. On such occasions, pending questions from the last meeting may be answered and prepared by the research expert for the next meeting.

Once the annotation is finished, the case is marked accordingly. Depending on the urgency and the number of cases in the backlog, the patient is subsequently scheduled for one of the upcoming MTB meetings. The treating physician has the option to receive notifications to know when his/her attendance is required: one is sent upon case creation, another one as soon as the patient is scheduled. Whenever the maximum number of patients per meeting is reached, a summary of scheduled cases is sent to all meeting attendees in advance to give them the option for preparation. Thus, all attendees can actively participate in the discussion during the MTB meeting.

In preparation for the meeting, a presentation screen is generated from a template. The template comprises case-specific data such as mutation burden, tumor signatures, functional variants, pathways, and treatment categories, selected annotations including drugs, respective evidences from the primary literature and clinical studies, and a prioritized list of treatment recommendations. Patient-specific data items relevant for the introduction and discussion of the case may be selected and added.

#### MTB Meeting

**Fig. 4:**
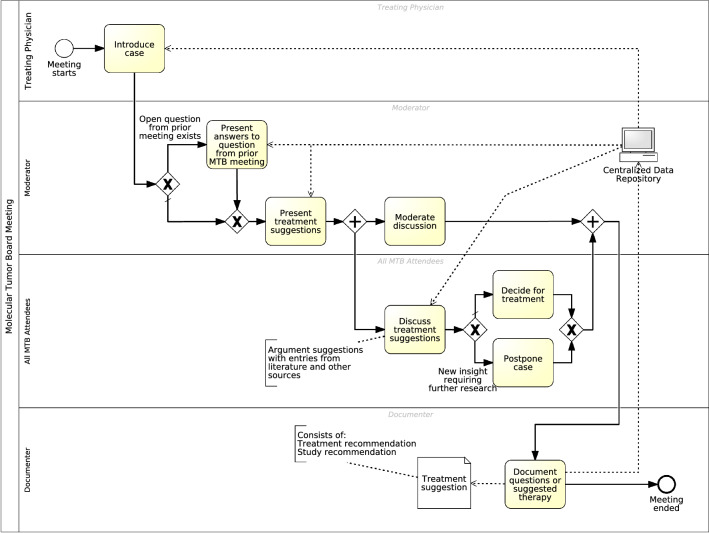
Process model for MTB meeting. During the meeting process, a patient case is introduced, and its medical history is presented briefly. Consequently, the moderator starts to follow up on any open questions from the last MTB meeting. Next, the moderator presents a prioritized list of treatment suggestions and available studies, including eligibility criteria and location. Afterwards, all attendees are guided through the discussion and are invited by the moderator to comment on the case and provide more suggestions that influence the treatment decision. Successively, the group agrees on the most appropriate treatment recommendation, which will be documented

The MTB meeting process is depicted in Fig. 4, which involves the treating physician, moderator, all MTB attendees, and a dedicated person for documentation. As in our observations, the aforementioned MTB research expert can also fulfill the moderator’s role. The MTB attendees are medical professionals with complementary expertise so that they can jointly propose a suitable treatment option. Typical professions include clinicians, representing all relevant specialisms, molecular biologists, pathologists, geneticists, and bioinformaticians [5, 61].

The number of MTB meetings per week shall scale with the number of cases and will become part of the regular TB meetings in the future. This allows us to have a holistic view on the patient’s condition and to make data-driven treatment decisions from the beginning on. Data silos shall be replaced by a centralized data repository to support fast access to both meeting agenda and de-identified patient data along the meeting through their personal devices.

Either the moderator or the treating physician or a representative introduces the patient case by briefly presenting the medical anamnesis. Consequently, the moderator starts to follow-up on any open questions from the last MTB meeting. The moderator continues to present a prioritized list of treatment suggestions and available studies including eligibility criteria and location. Afterwards, all attendees are guided through the discussion and are invited by the moderator to comment on the case as well as to provide more suggestions that influence the treatment recommendation.

Since the preparation process enables each participant to prepare themselves in advance, the discussion is expected to be more engaging. Although cases should not be postponed due to missing data, in some occasions of postponements, the case shall by digitally tagged including reason and open questions eliminating any paper notes. Successively, the group agrees on the most appropriate treatment recommendation, which is documented.

In contrast to our observations, we assign a dedicated person to the user role of the documenter. The documenter notes down therapy recommendations, clinical trials and additional remarks, live and transparently on the presentation screen visible to the attendees. Upon completion of the structured documentation, a report is automatically generated and provided to the treating physician.

#### MTB Follow-Up

Regularly conducted follow-ups, e.g. after three to six months, evaluate the adherence of the therapy to the MTB recommendation and help to analyze the efficacy of treatment recommendations as evidence for future cases. Personal notifications invite the patient to the hospital to be reexamined, which are automatically sent after a certain period of time.

During a regular follow-up visit to the clinic, an imaging of the tumor is performed and evaluated. This goes along with a doctor’s appointment and a clinical examination, where information on general and nutritional condition, current medication, laboratory results, other relevant diseases, comorbidities, symptoms and side effects is collected.

If the patient is repeatedly not responding to follow-up invitations, one option is to query—ideally in an automatic fashion—local or federal state cancer registries to receive further information about the patient’s follow-up status from external clinics or physicians. If such cancer registries are not available, at least local registration offices of the patient’s hometown should be contacted for information about the patient’s vital status and to check whether the patient has moved to a new address.

More details may be received directly from the patient or his local practitioner. For this purpose, the patient must give consent upon hospital admission, allowing the hospital to contact her/him or the local practitioner.

The received insights shall be linked to the patient case to enrich the overall patient data within the centralized data repository. Given a future case and any identified historic similar patients, treatment options can be assessed based on existing statistics about the efficacy of previous recommendations.

### Relevant Case Information

Today, experts in TBs rely on a multitude of diverse information to determine the most appropriate treatment decision, stored in HIS as depicted in Fig. 3. In the future, additional data sources that need to be available for the MTB meeting as well as data that is generated during an MTB session are outlined.

In addition to the molecular characterization of genetic variants, other clinical parameters such as the medical history and the current general condition are essential. They allow assessing identified treatment options according to previous treatments, potential side effects, and pros and cons given the specific situation of a patient.

Detailed clinical, pathological, and radiological information at least from the latest examinations should be provided to evaluate the current status of the tumor. For example, this includes results from clinical examinations of the patient including general condition, nutritional condition, current medication, laboratory results, relevant diseases, comorbidities, current symptoms and side effects. Pathological information encompasses parameters like histology, current tumor staging, and additional tumor entity dependent classifications. From a radiological point of view, longitudinal imaging data is relevant. Time points include the first diagnosis date, before and after treatments and re-stagings. At each time point, several radiologic parameters are reported, e.g., primary tumor, recurrences, metastases. This includes localizations, diameters and details about the imaging technique that has been performed, like the contrast agent, imaging phase or Magnetic Resonance Imaging (MRI) weighting. Especially, follow-up imaging is important to detect early relapse after treatment.

Furthermore, each MTB session results in new insights that have to be stored and linked for later reuse. During the MTB preparation phase, the participants may comment on available data. During the MTB meeting, the comments can be discussed and additional comments may be added. Most prominently, the tumor board recommendation for each case discussed has to be written, signed, and transferred back to HIS. The tumor board recommendation includes all relevant clinical, pathological, radiological, and molecular information but also relevant clinical trials, literature, and information about similar cases that were determining factors for the recommendation.

Prior to the follow-up meeting with the patient, a current imaging of tumor sites is routinely acquired and evaluated to assess therapy success or relapses. Additionally, a clinical examination is conducted to acquire updated data on general condition, nutritional condition, current medication, laboratory results, relevant diseases, comorbidities, current symptoms, and medication side effects. Especially information about the current treatment of the patient after MTB recommendation is of interest, since not all recommendations may have been realized. As a last resort, the vital status can be queried from cancer registries or local registration offices. Based on the follow-up information, statistics about individual recommendation acceptance, progression-free survival times, and potential toxicities are created as an important feedback on therapy efficiency and to improve treatment of future cases.

### Software Prototype

**Fig. 5:**
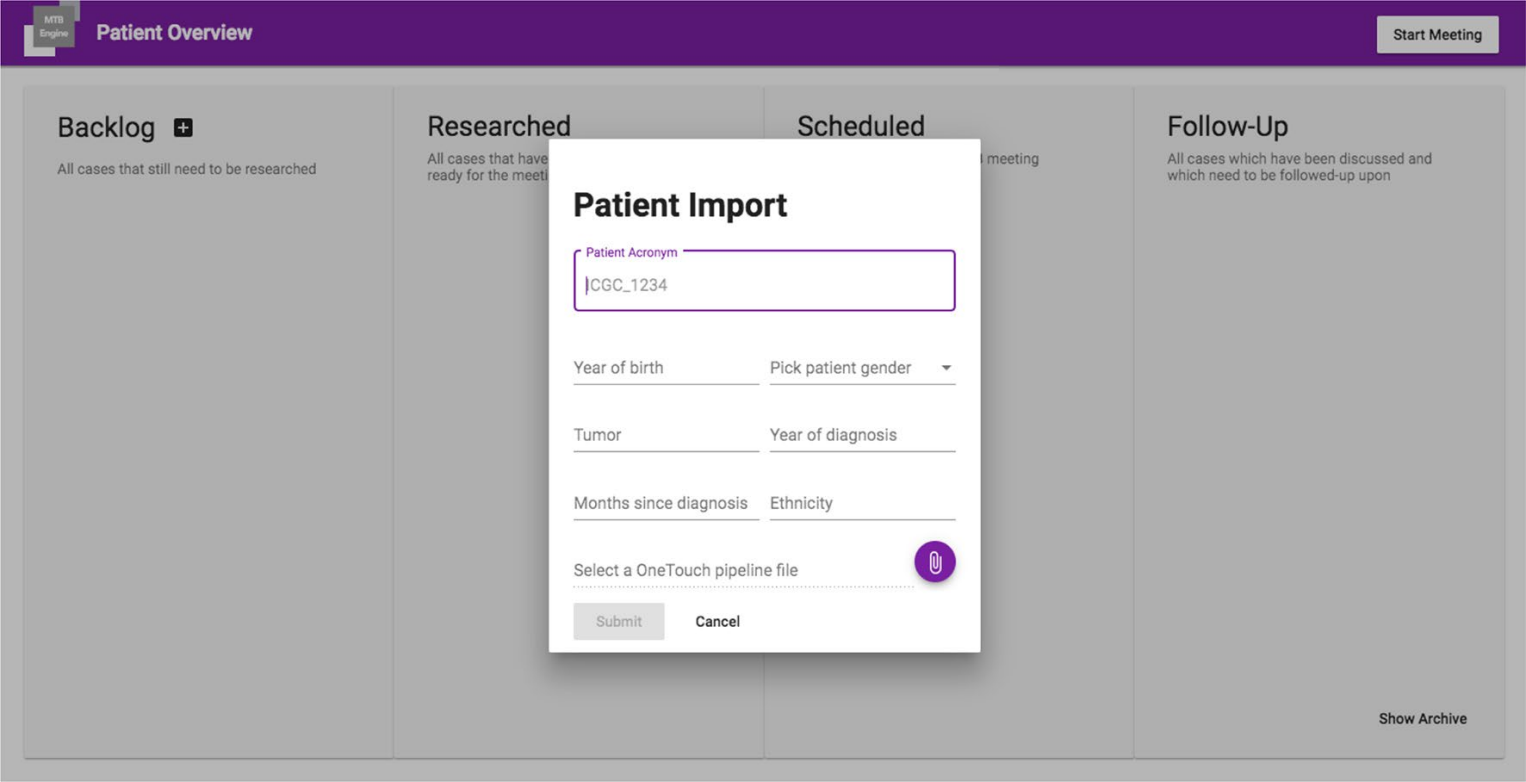
Screenshot showing a dialogue to manually create a case by providing an identifier, demographics and diagnosis in MTB Assist. These are the minimal required clinical data, as well as the minimal set of identifying information. Patient names are not stored or displayed in the software, such that cases can safely be discussed across multiple sites. Sequencing data can be uploaded in a standardized data format

**Fig. 6:**
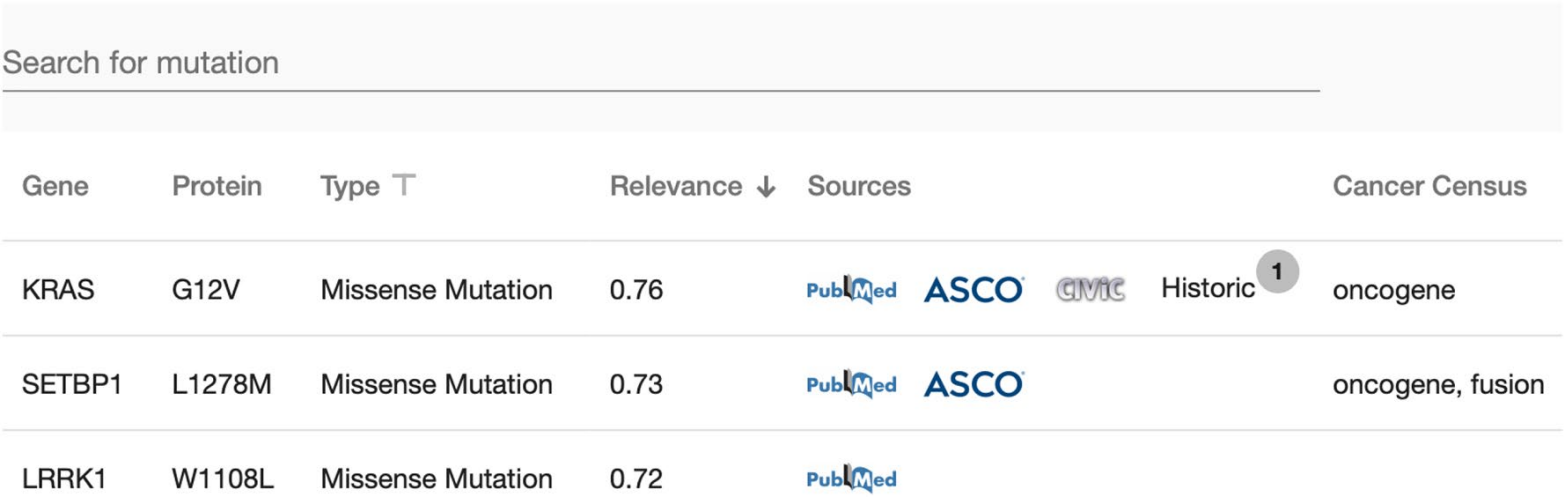
Variant view for a selected case. Amongst others, it depicts altered genes, e.g. KRAS, SETBP1, LRRK1, type of variant, relevance, primary sources, similar cases, and the role of the gene according to the Cancer Gene Census database. With this information, variants can be filtered, sorted, and prioritized for further manual annotation

**Fig. 7:**
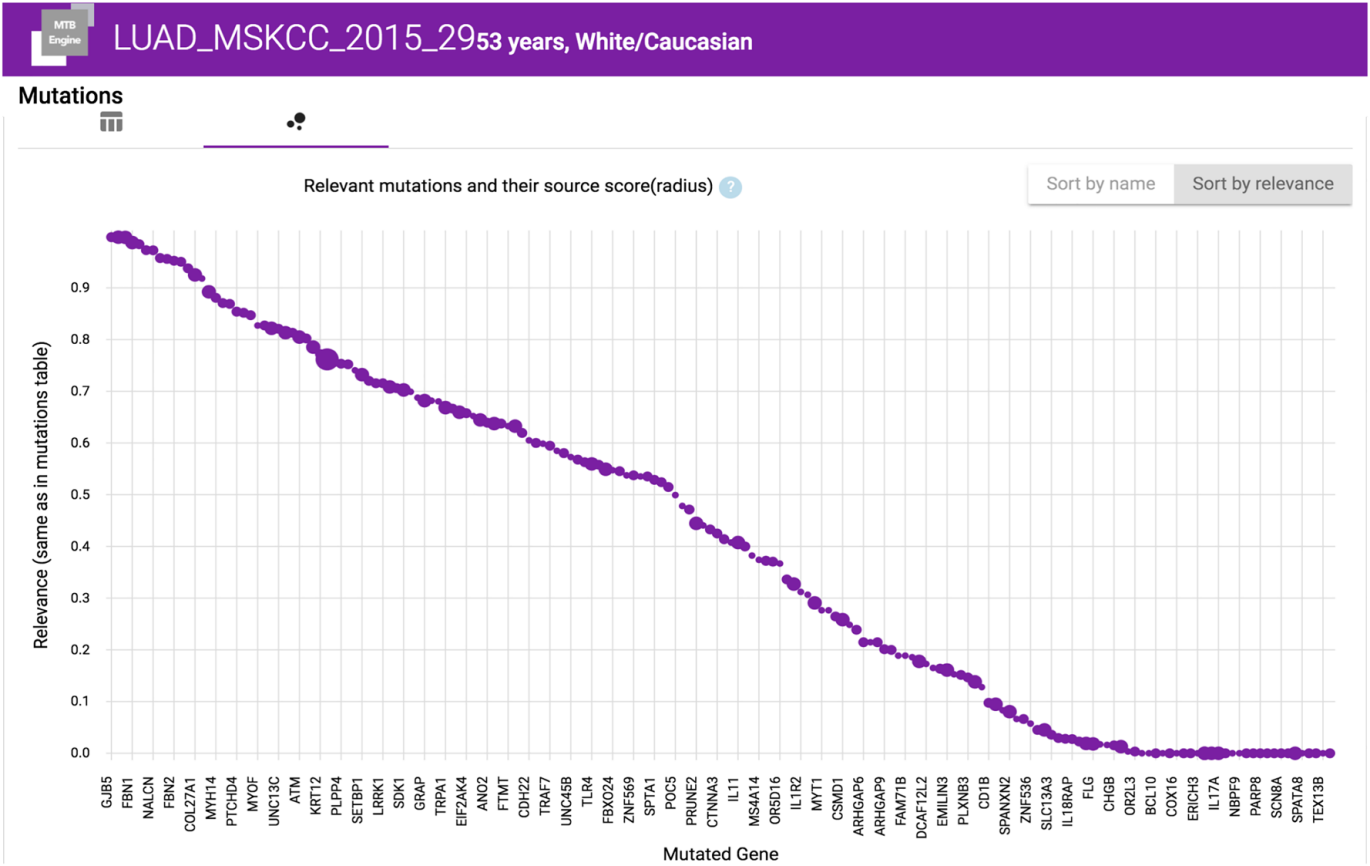
Screenshot showing functional genetic variants (x-axis) sorted by their calculated relevance score (y-axis)x. The graph shows the score that is calculated by the appearance of gene mutations in multiple sources: to account for source quality, we put a higher weight on specific sources, making those mutations stand out more by appearing higher up in the list and having a bigger circle inside the diagram. We value sources from CIViC higher since entries are most likely created by MTB research experts or equivalent professionals. Historical cases are valued highest since entries stem from known experts in the field

**Fig. 8:**
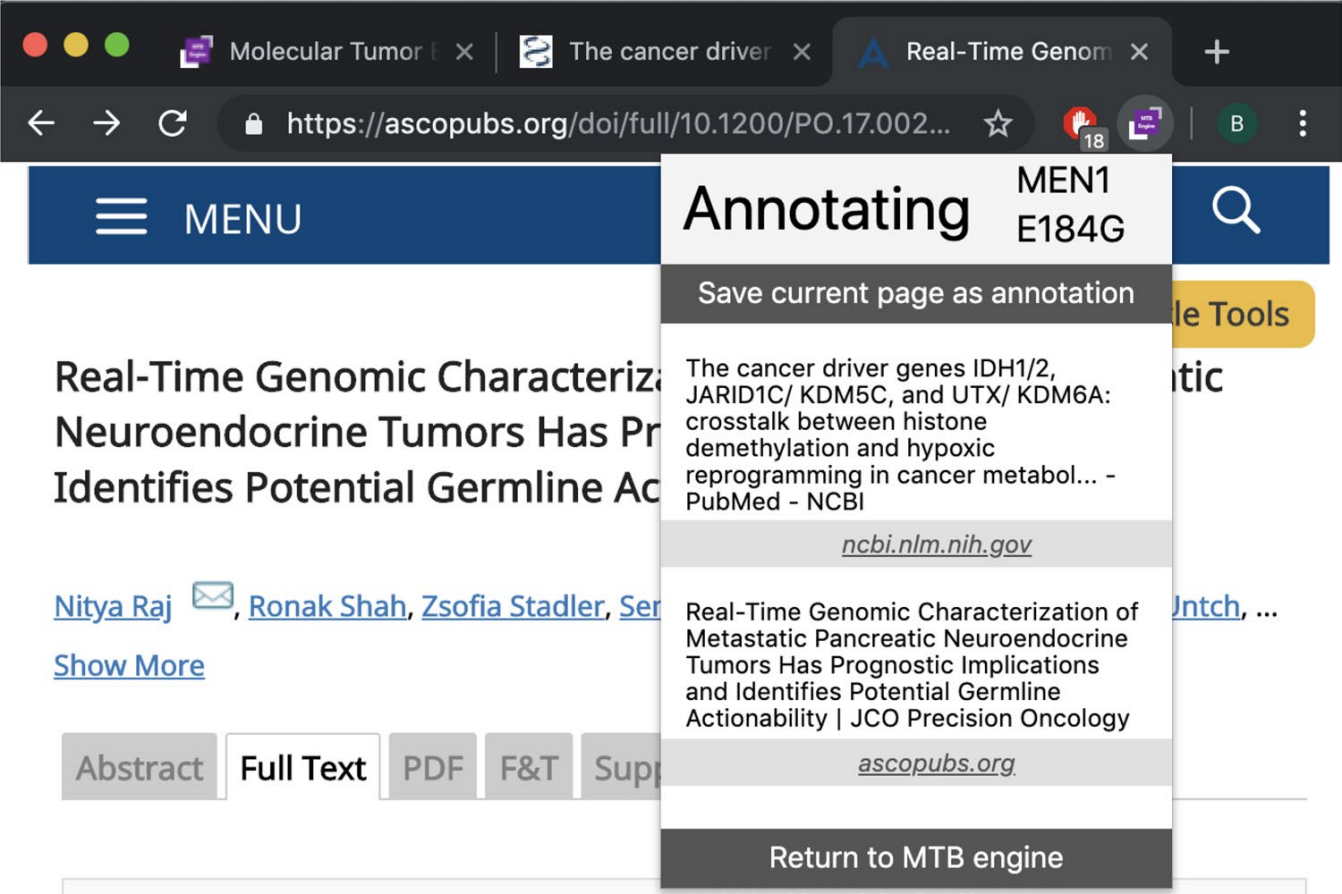
The browser plugin assists in keeping track of annotations from arbitrary sources, which matches clinical practice. As most relevant knowledge bases have either no or non-standardized APIs, such an integration on the frontend level allows linking cases to evidence from any Web-based data source

**Fig. 9:**
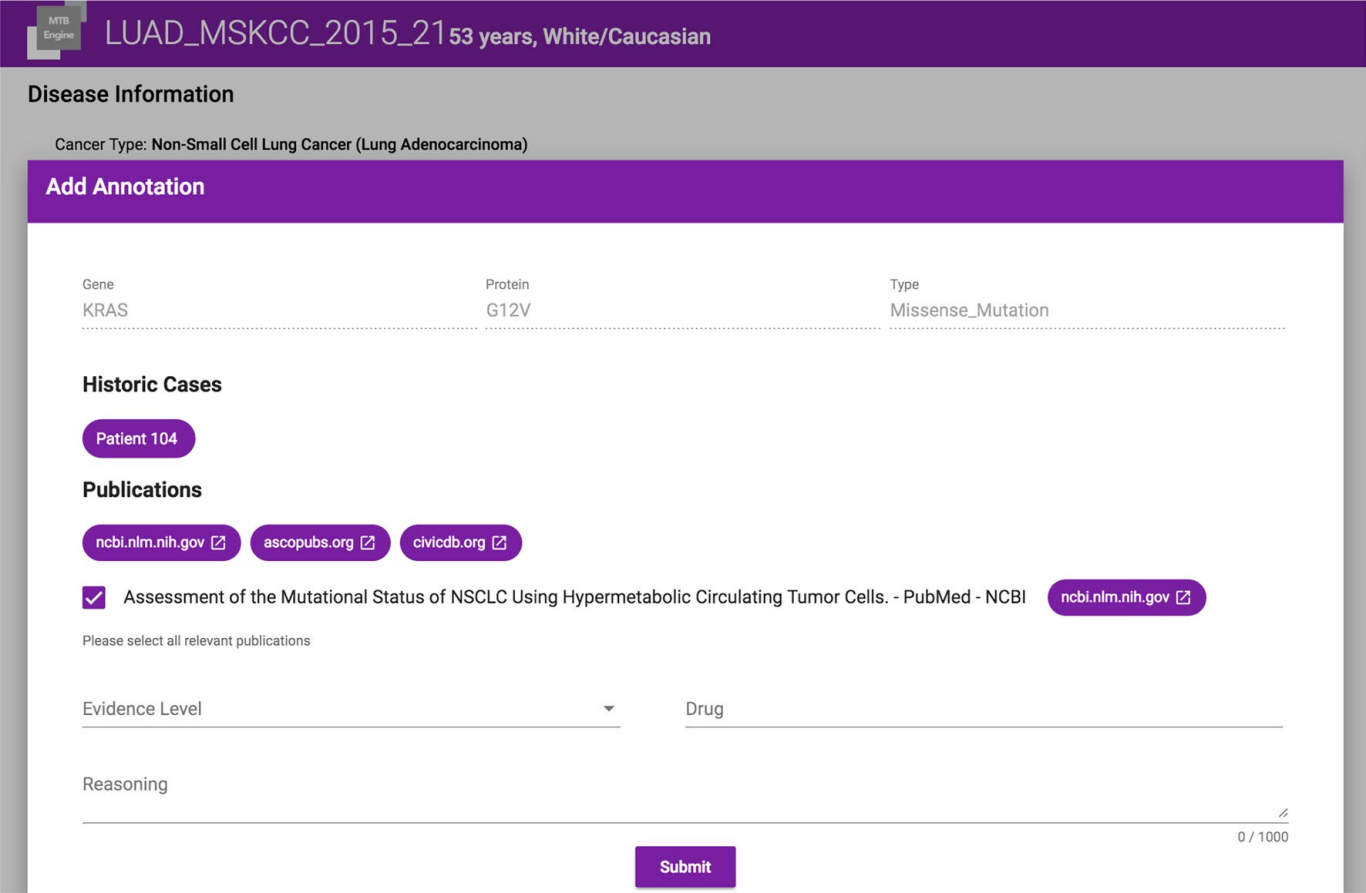
A screenshot of the annotation screen. The upper part shows the selected mutation. The middle part depicts links to similar historic cases in the central data repository, literature references and hyperlinks to data sources containing at least one entry for the given mutation. The lower part shows the assigned clinical evidence level and the resulting treatment recommendation. This information is available to all MTB participants prior to the meeting, fostering collaboration and exchange of knowledge among MTB experts, even across MTB meetings through access to historic cases

The clinical processes identified in Sect. “Clinical Process Model” are supported by our prototype MTB Assist to address software requirements outlined in Sect. “Software Requirements” as follows:

*Import of Case Data: * Case data can be added to the system either manually as depicted in Fig. 5 or by querying the local clinical data repository, e.g. the local MeDIC (F2). Case-related data is stored in an IMDB to enable real-time data analysis. Each case is represented as a card and becomes visible in the first column of the case overview depicted in Fig. 1.

*Case Overview: * The dashboard depicted in Fig. 1 implements the Kanban methodology to manage the current state of each case in the MTB process [62–64]. Kanban is an agile method that improves communication and coordination, visualizes the work in progress, and reduces process times. Nowadays, it is also used in agile software engineering to improve communication and coordination, to visualize the work in progress, and to reduce process times: properties that we want to adopt for MTB management as well. Cases are arranged in columns, namely *backlog*, *researched*, *scheduled* and *follow-up*, that help MTB research experts to organize their work (F3). The backlog shows cases that were created but have not been worked on, whereas researched cases have already been annotated. Cases with state *scheduled* will be presented in the next tumor board. As soon as a recommendation has been given, the case moves to a *follow-up* state to remind the research expert to obtain follow-up information, e.g., whether the patient received the proposed therapy, medication, doses, and whether side effects occurred.

*Variants View and Literature Sources: * All functional genetic variants, gene locations, and affected proteins are shown in the view outlined in Fig. 6. Amongst others, the following knowledge sources are available: PubMed, ASCO, CIViC as well as data from historical cases. Thus, manual research time can be reduced (F6).

*Relevance Scores: * Mutations can be sorted by a relevance score calculated using functional analysis through hidden Markov models obtained by VarSome [65]. The score indicates the functional effects of protein missense mutations (F5). Further scores may be implemented [66]. Interactive figures help to assess driver mutations as depicted in Fig. 7.

*Similar Cases: * Historical data enables reusing existing clinical knowledge, also from other hospitals. We defined three levels of case similarity, addressing F7: Weak similarity is given by case characteristics such as age, gender, diagnosis. In addition, similar cases can be matched using molecular genetics, e.g., detected alterations affecting the same loci or genes. The third level of similarity is given by combining all available structured patient and tumor characteristics through the use of Artificial Intelligence (AI) algorithms [67]. Although the use of AI-trained models might create promising results, the explainability of the results might be challenging depending on the incorporated model type.

*Annotation Plugin: * Our tool either supports the annotator through a browser plugin for Google Chrome as depicted in Fig. 8 or a structured form as depicted in Fig. 9, which enables manual enrichment by meta-data including medication, evidence level and further comments (F4). Annotations appear as treatment recommendation on the case’s details screen.

*Detail View: * Today, clinical, pathological, and radiological data are available per case for traditional TBs. This data is displayed next to the variant view, allowing to assess whether a identified treatment is recommendable to a patient and in order to prepare the presentation in which the patient’s medical history is revisited (F2).

*Presentation View: * Once a case has been annotated, it is moved to the Kanban board *researched*. If a case shall be discussed in the next meeting, it will be moved to the board *scheduled*, from where it can be selected for presentation by a single click.

All cases can be opened in presentation mode in favor of manual-created MS PowerPoint slides addressing F9.

In preparation of an upcoming MTB session, the research expert may request feedback from individual participants prior to the meeting (F8).

*Documentation:* The meeting screen includes a documentation tab, where the treatment recommendation can be documented together with the evidence level, variants, drugs, and available clinical trials (F10). An additional free-text field allows noting down further comments.

*Access Control: * We have implemented an access control system, which allows assigning subjects and participants to groups, limiting access to individual case data (F1).

## Evaluation

In the following, we evaluate our findings and discuss their applicability with regard to multi-site virtual MTBs.

Firstly, we need to consider how to establish the required clinical processes for such MTBs. Our defined MTB process models presented in Sect. “Clinical Process Model” were created and validated together with subject-matter experts from multiple German hospitals. Thus, they built upon best practices from hospitals conducting MTBs. Furthermore, subject-matter experts were able to provide feedback on how to adapt these processes. We found out that for some hospitals there will be selected specifics. Therefore, our clinical process models aim to be flexible and open to include these hospital specifics as well.

The models are open to participants from remote entities via a conferencing system. Inter-institutional information exchange is the basis for the identification of similar historic cases. Therefore, the search for selected non-identifiable attributes should be supported, such as gender, age group, primary diagnoses, selected genetic changes.

We consider the combination and sharing of clinical knowledge between medical experts—even across institutional borders—as an enabler for a more cooperative decision-making process in oncology. In addition, the idea of displaying selected data from similar cases has been perceived as a unique advantage because it is not available in clinical practice, yet. The ability to automatically turn assembled case data into a screen presentation was named as an essential feature. It releases medical professionals from composing slides using Microsoft PowerPoint and making additional time for medical investigation available.

Interviews with subject-matter experts revealed that the quality of an MTB highly depends on the work of the MTB research expert. By leveraging our software tool MTB Assist, the burden on they are reduced in the long term because the system grows through multi-center use and allows the combination of annotation from all sites. Thus, we expect that the most time-consuming step, the research for patient-specific annotations, will reduce over time.

Our tool forms the foundation for building up a multi-site knowledge base, which contains, amongst others, point-of-care data, annotations for molecular variants, assessed treatment recommendations, and clinical trials enriched by selected clinical data. We consider it as a management support system that helps to implement standardized MTB processes.

The feedback from medical professionals revealed that our proposed MTB Assist provides suitable IT-aided process support for the preparation and conduction of tumor boards. For example, the linkage to primary annotation knowledge bases supports reproducible decision-making. Accessing public annotations from distributed databases for genetic variants via a single user interface was considered time-saving. Our interview partners highly appreciated the modular fashion of source inclusion for the annotation. However, the correct selection of sources remains challenging, and comprehensive tool support is missing [9]. The drug auto-complete option in the annotation view was considered helpful because it avoids inconsistencies. However, it lists only approved medications, and it does not contain recent drug candidates available from clinical trials. During our interviews, we found out that Microsoft Internet Explorer and Mozilla Firefox are the most common Web browsers in hospitals. However, our plugin to link literature builds on specific functionality provided by Chrome browser only. As a result, the recommendation for hospital IT is to enable the use of alternative Internet browsers to enable use of instant linkage of literature to our software tool. Nonetheless, literature references can also be added manually via a structured form. However, the manual literature linkage is more time-consuming and may introduce typos compared to the instant linkage provided by our plugin.

## Discussion and Limitations

In Sect. “Observations and Current Limitations”, we identified challenges in current implementations of MTBs. Our software prototype addresses the majority of these challenges, while others still have to be considered open problems that need to be addressed by the research community.

The first set of identified challenges are related to the tedious manual variant annotation process (C1-C5). With our approach, we attempt to streamline this process through a browser plugin and access to historic cases within the central data repository. Yet, it does not free the MTB research expert from manually validating the information from disparate knowledge sources. A comprehensive integrated knowledge base, which should comprise historic cases from the within the system, the scientific literature, as well as case reports and clinical trial results from publicly available data sources, is currently not available [9].

As a management tool, our software addresses the challenges related to non-standardized processes, a lack of structure and integration of clinical data (C6-C8). Structured, digitized documentation is another challenge in current MTB implementations (C11), which is enabled by our software, but will require deeper integration with hospital-specific, downstream software systems for tumor documentation.

Since all participants have access to the prepared cases, we established a basis for a shared understanding and discussions in the MTB meeting (C9). While this is a substantial improvement over current solutions, there is currently no further support for discussions in the meeting. Enabling participants to comment on cases during preparation would enable even deeper collaboration.

The last challenge is related to data privacy and access rights (C10). Currently, data privacy and authorization is established by avoiding any identifiable information within the software (only pseudonyms are used) and assigning user accounts to different user groups, typically a single hospital. When more clinical data is integrated, a more elaborate management of access rights will be required.

## Conclusion and Outlook

With our incorporated DT methodology, we were able to assess current limitations in conducting multi-site MTBs. We identified current state-of-the-art processes and defined clinical process models using BPMN to facilitate the fast implementation of MTBs. As MTBs require tool support we designed a software support tool that addresses crucial management challenges. Our contribution includes the clinical process models modeled using the BPMN version 2.0 standard. Thus, our results can be used as a blueprint for the standardized implementation of MTBs in hospitals across Germany and other countries. Finally, we demonstrate how software tools like our proposed MTB planning software tool can support the IT-aided clinical process. Our software prototype addresses oncologists’ requirements and combines fragmented knowledge. The Web-based software tool supports more efficient and effective treatment planning. Our contributions open up opportunities for future work regarding knowledge integration, data sharing, and interaction with up- and downstream clinical software applications.

In the following, we share possible future research directions identified throughout the course of analyzing the clinical processes and state-of-the-art work in conducting MTBs.

*Systems Integration: * In this work, we have focused on the process of the MTB preparation and meeting. Each clinical process is embedded in a complex patient journey and has interfaces to existing clinical processes and clinical software systems. While our MTB Assist prototype can be used in a standalone manner, integration with existing infrastructure is key for its real-world use.

Clinical data of the patient is already collected in electronic health records or data repositories, which are established in the course of the MII [14]. A comprehensive view of this clinical data for the preparing MTB research expert is necessary to prioritize treatment recommendations [68]. In the future, our tool should also support the selection and concise presentation of relevant subsets of clinical data for discussion in the MTB meeting.

Integration of our software with heterogeneous sequencing pipelines is another challenge to be addressed, in particular when more biological layers are added to the analysis. Standardized interfaces to sequencing data would allow to streamline the variant annotation process. Instead of developing a de-novo tool, prior work has proposed to extend existing widely used software such as cBioPortal, which could simplify adoption [11].

*Documentation and Follow-Up: * A critical bottleneck in current MTBs is the re-integration of treatment suggestions into hospital information and tumor documentation systems to enable a standardized follow-up.

To enhance this feature of our software, more user research will need to be conducted to generalize needed information for documentation, auto-completion of documentation items, cleansing of documentation, and structured storage of data. Annotators should also be enabled to search in the documentation. Eventually, our system is envisioned to integrate with existing clinical documentation systems such as the GTDS for the retrieval of existing documentation [58].

*Integration with Clinical Guidelines: * At the moment, MTBs are used as a research platform to generate evidence for treatments beyond the currently published clinical guidance. A process-oriented view combining the implemented MTB processes with recommendations from clinical guidelines would allow to better integrate these parts of the patient journey. On the one hand, there is currently no automated way to verify adherence to guidelines in the first place, while inclusion into MTBs is nowadays often only considered after guideline-recommended treatments have failed. On the other hand, evidence generated in MTBs should ultimately inform recommended clinical practice.

*Sharing Knowledge with the Community: * Referring to the reuse of knowledge, the annotated mutations and treatment suggestions are currently stored and accessed within our MTB Assist system. To enable timely sharing of generated evidence, it will be important to enable automated submission of findings to community platforms, such as CIViC [50]. Furthermore, the match of similar cases can also be elaborated, where the annotator has the quick access to past cases similar to the patient currently researched on. This includes not only cases within our system and public knowledge bases, but also published case reports in PubMed. Automated information extraction from these case reports through Natural Language Processing (NLP) would be required to tap into this rich source of information on a large scale [69, 70]. Beyond getting access to similar case data at all, it is necessary to define clinically meaningful similarity measures of patients based on the genetic profile of tumor samples and clinical history.

## Data Availability

There are no shared data available with this publication.
